# DYRK1A in the physiology and pathology of the neuron-astrocyte axis

**DOI:** 10.3389/fnins.2025.1626062

**Published:** 2025-10-20

**Authors:** Pablo Cisternas, Jiyoon Kim, Brandon Ashfeld, Jeremiah Zartman

**Affiliations:** ^1^Department of Chemical and Biomolecular Engineering, University of Notre Dame, Notre Dame, IN, United States; ^2^Department of Biological Sciences, University of Notre Dame, Notre Dame, IN, United States; ^3^Department of Chemistry and Biochemistry, University of Notre Dame, Notre Dame, IN, United States

**Keywords:** DYRK1A, gene dosage, neuron, astrocyte, Alzheimer’s disease

## Abstract

Dual-specificity tyrosine phosphorylation-regulated kinase 1A (DYRK1A) is a dosage-sensitive kinase with critical roles in the neuron-astrocyte axis. During brain development, DYRK1A ensures the proper number of differentiated neurons and astrocytes. In neurons, this DYRK1A regulates neuronal morphogenesis and synaptic transmission. However, its functions in astrocytes are not yet well defined, with limited evidence indicating roles in astrocyte reactivity and excitotoxicity. Due to trisomy 21, DYRK1A is overexpressed in individuals with Down syndrome (DS). This imbalance directly contributes to neuronal death and likely astrocyte pathology, accelerating the onset of Alzheimer’s disease (AD) in this population. Notably, DYRK1A overexpression also correlates with neurodegeneration and AD progression in elderly euploid adults. This correlation positions DYRK1A as a potential bridge between DS and AD, mechanistically connecting gene overdosage and neuropathology in both conditions. However, research on DYRK1A pathophysiology has primarily centered on neurons, leaving astrocytes largely understudied. Considering the vital neuroprotective functions of astrocytes, broadening DYRK1A research to encompass these cells presents an opportunity to uncover novel mechanisms contributing to the neurodegenerative process in AD. In this review, we highlight the physiology and pathology of DYRK1A in the neuron–astrocyte axis, analyzing its roles in neurons and positing hypothetical functions in astrocytes, with particular emphasis on the contribution of DYRK1A’s cell-specific overexpression to neurodegeneration and AD progression.

## 1 Introduction

**FIGURE 1:**
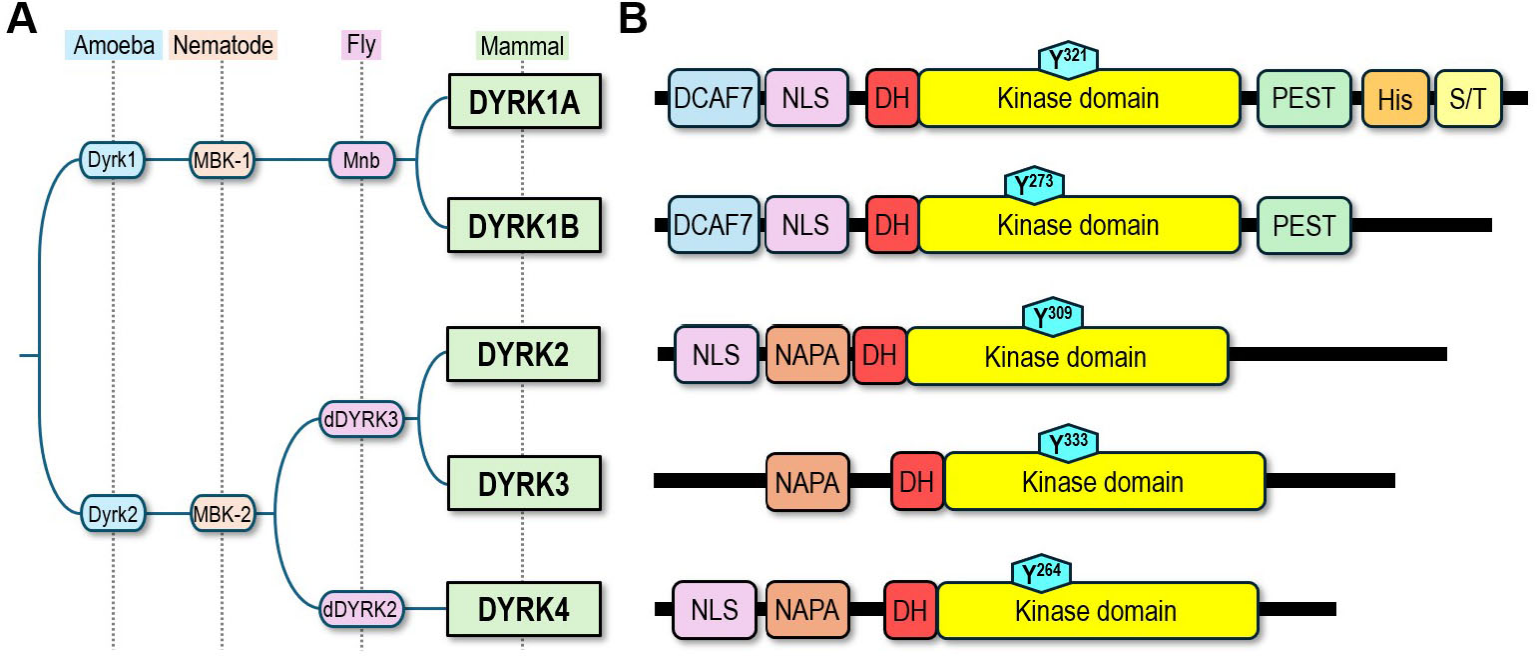
The DYRK subfamily of kinases. **(A)** DYRK orthologs in other eukaryotes (left) and subfamily members in mammals (right). **(B)** Structural organization of mammalian DYRKs, highlighting domains and autophosphorylation residue (cyan-colored hexagon). DCAF7, DCAF7-binding domain; NLS, nuclear localization signal; DH, DYRK homology box; PEST, protein degradation domain; His, histidine repeat; S/T, serine/threonine-rich motif; NAPA, autophosphorylation accessory region. Figure synthesized from Adayev et al. (2007), Aranda et al. (2011), Ashford et al. (2015), Deboever et al. (2022), Li et al. (2002), Papadopoulos et al. (2011), Park et al. (2009), Suanes-Cobos et al. (2025), Tandon et al. (2021), and Walte et al. (2013).

DYRK1A is a multifunctional regulatory kinase operating in the physiology and pathology of the neuron–astrocyte axis, the structural and functional unit assembled by continuously interacting neurons and astrocytes (Benarroch, 2005). A member of the DYRK subfamily of kinases (Figure 1A), DYRK1A comprises multiple structural regions, of which the kinase domain is the most conserved among DYRKs (Aranda et al., 2011; Figure 1B). To achieve full kinase activity, DYRK1A autophosphorylates at a conserved tyrosine residue within its activation loop (Himpel et al., 2001; Figure 1B, cyan hexagon) while transitioning through a short-lived intermediate state (Lochhead et al., 2005), yielding the mature form of DYRK1A with immediate capacity for phosphorylating its target proteins in serine or threonine residues (Becker and Sippl, 2011; Park et al., 2009). DYRK1A’s activity can be further modulated—enhanced through calpain-dependent proteolytic truncation (Jin et al., 2015) or inhibited by binding partners such as FAM53C (Miyata and Nishida, 2023) or the 14-3-3 protein (Kim et al., 2004).

**FIGURE 2:**
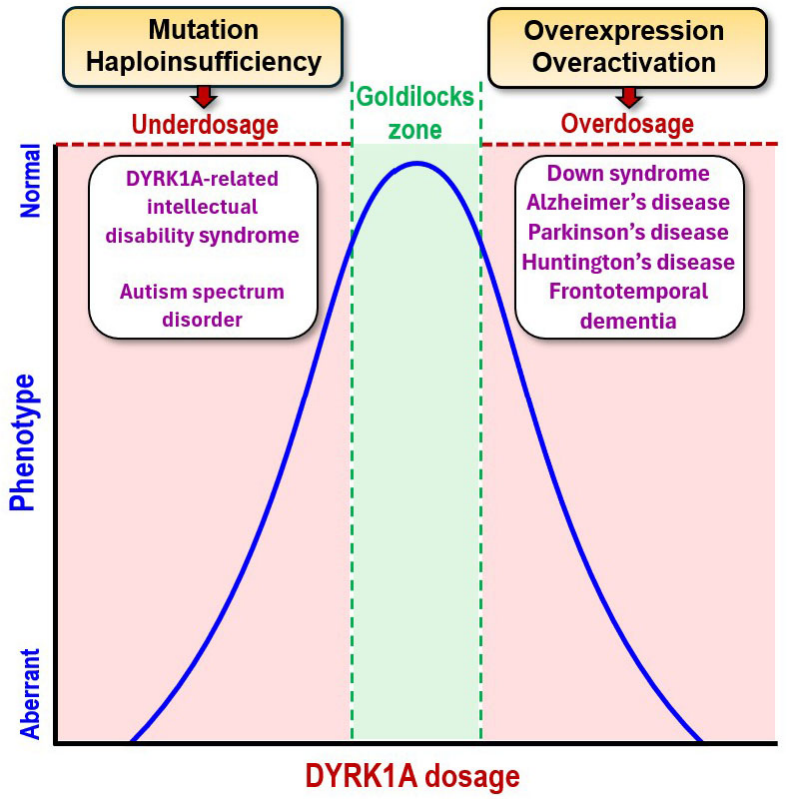
DYRK1A dosage imbalance is implicated in the onset of multiple neurological conditions. For appropriate downstream effects (blue), DYRK1A dosage (red) must be tightly regulated within an optimal ‘Goldilocks zone’ (green). DYRK1A gene mutation or haploinsufficiency reduces DYRK1A dosage and contributes to DYRK1A-related intellectual disability syndrome (MRD7) and autism spectrum disorder (ASD). Conversely, DYRK1A gene overexpression/overactivation increases DYRK1A dosage, driving the pathogenesis of Down syndrome (DS), Alzheimer’s disease (AD), Parkinson’s disease (PD), Huntington’s disease (HD), and frontotemporal dementia (FTD). Figure inspired by Ananthapadmanabhan et al. (2023), Arbones et al. (2019), Deboever et al. (2022), and Duchon and Herault (2016).

Given that DYRK1A is constitutively active, its downstream effects are determined by tight dosage control. Accordingly, DYRK1A dosage imbalance is consistently detrimental and is linked to the appearance of a broad spectrum of neurological disorders (Ananthapadmanabhan et al., 2023; Deboever et al., 2022; Duchon and Herault, 2016; Tian et al., 2019; Figure 2). In humans, the DYRK1A gene lies within the DS critical region of chromosome 21, which is triplicated in individuals with DS due to trisomy 21. This genetic alteration leads to DYRK1A overexpression (Lowe et al., 2019; Wegiel et al., 2008), disrupting neurogenesis and gliogenesis during brain development, which contributes to the neurological impairments exhibited by DS individuals (Guedj et al., 2012; Stagni and Bartesaghi, 2022). Importantly, DYRK1A overdosage accelerates the onset of AD in DS adults (Liu et al., 2008; Lowe et al., 2019; Wegiel et al., 2008, 2011b), lowering the mean age of appearance to 50–54 years old (Larsen et al., 2024) and increasing its prevalence to 90–100% in this population (Fortea et al., 2021). The brains of these individuals show elevated levels of amyloid-beta (Aβ) plaques and neurofibrillary tangles (NFTs), the pathological hallmarks of AD (Cipriani et al., 2018). Most significantly, DYRK1A overexpression is also observed in sporadic AD patients despite an euploid background, resulting in equivalent neuropathological manifestations as those seen in adult DS cases (Ferrer et al., 2005; Kimura et al., 2007), although the timing and localization of this imbalance can differ between these two conditions (Wegiel et al., 2008). Nevertheless, the same overabundance that drives the neuropathological process in DS also promotes it in AD, where DYRK1A provides a direct mechanistic link between gene overdosage and disease progression (Martínez-Cué and Rueda, 2020; Wegiel et al., 2011a).

**FIGURE 3:**
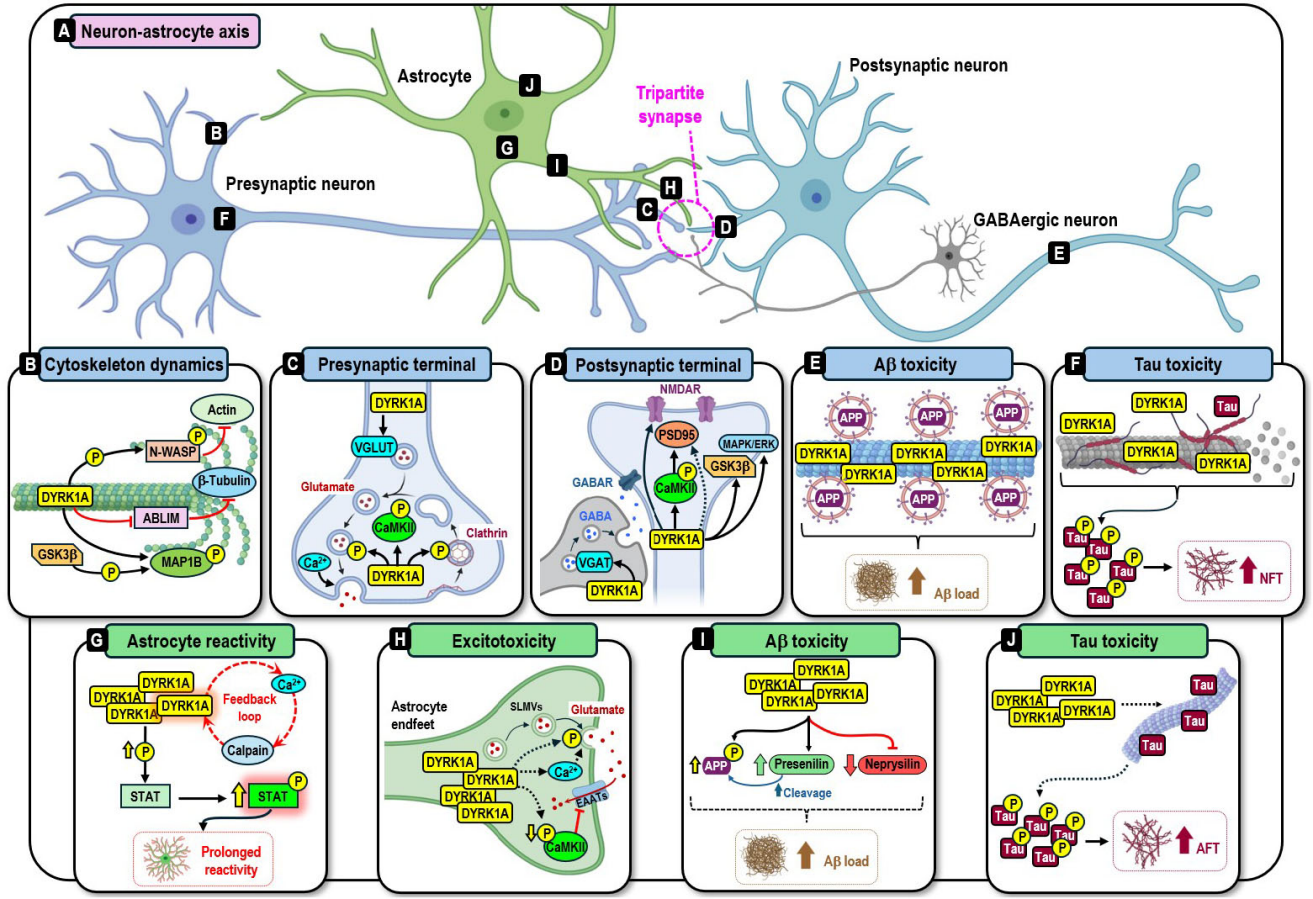
The diverse functions of DYRK1A at the neuron-astrocyte axis **(A)**. In neurons, DYRK1A regulates cytoskeletal dynamics and morphogenesis **(B)**. At the tripartite synapse, DYRK1A tunes synaptic transmission in both pre- **(C)** and post-synaptic terminals **(D)**. On the other side, DYRK1A overexpression leads to Aβ pathology **(E)** and tau toxicity **(F)**, which promote neuronal death. In astrocytes, DYRK1A modulates astrocyte reactivity **(G)** and potentially mediates glutamate excitotoxicity **(H)**. However, astrocytic DYRK1A overdosage could trigger Aβ **(I)** and tau pathology **(J)** and compromise the astrocyte’s neuroprotective functions.

Importantly, DYRK1A carries out multiple roles in the neuron–astrocyte axis (Figure 3A). In neurons, DYRK1A regulates neuronal morphogenesis (Dang et al., 2018; Manubens-Gil et al., 2023; Martinez de Lagran et al., 2012) and tunes synaptic transmission at both pre- and postsynaptic terminals of the tripartite synapse (Arbones et al., 2019; Grau et al., 2014; Souchet et al., 2014; Thomazeau et al., 2014; Wu et al., 2022). However, DYRK1A overdosage causes an aberrant interaction with its targets, activating several neurotoxic pathways involving Aβ (Fernandez Bessone et al., 2022; Ryoo et al., 2008; Wegiel et al., 2011a) and hyperphosphorylated tau (Gendron and Petrucelli, 2009; Kumar et al., 2015; Liu et al., 2008; Ryoo et al., 2007; Wegiel et al., 2008). In comparison, the function of DYRK1A in astrocytes remains poorly understood, with limited evidence suggesting an involvement in the regulation of the astrocyte’s neuroprotective capacity by modulating astrocyte reactivity (Ceyzériat et al., 2018; Gong et al., 2019; Kurabayashi et al., 2015; Lee et al., 2020; Melchior et al., 2019; Ryu et al., 2010; Souchet et al., 2019; Yamamoto et al., 2017) and glutamate excitotoxicity (Li et al., 2011). This is critical since astrocytes play a crucial role in maintaining neuronal homeostasis and survival (Kim et al., 2019; Sofroniew and Vinters, 2010). Without astrocytic support, neurons lose their ability to sustain synaptic activity and maintain long-term viability, promoting neurodegeneration (Ding et al., 2021; Verkhratsky et al., 2023). Considering the vital protective role of astrocytes, their contribution to neuronal death when pathologically impaired (Huang et al., 2025), and their sensitivity to the pathological events of AD (Arranz and De Strooper, 2019), expanding the study of DYRK1A’s pathophysiology to include astrocytes holds the potential to reveal novel mechanisms contributing to the neurodegenerative process in AD.

In this review, we examine the multifaceted roles of DYRK1A in the neuron–astrocyte axis. We first outline its functions in neurons, from its interactions with cytoskeletal regulators to control neuronal morphogenesis to the modulation of synaptic transmission and neuronal plasticity. Next, we discuss the pathological outcomes of DYRK1A overexpression in neurons. Then, we integrate the available scattered evidence to characterize the functions of DYRK1A in astrocytes, positing its interactions to modulate astrocyte reactivity and glutamate excitotoxicity. Accordingly, DYRK1A overexpression potentially impairs the neuroprotective role of astrocytes by disrupting these key processes or triggering novel Aβ/tau-dependent pathological mechanisms.

## 2 DYRK1A in the physiology and pathology of the neuron

During neurogenesis, DYRK1A is consistently expressed throughout the neuronal lineage. DYRK1A halts the proliferation and triggers the differentiation of neuronal progenitor cells (NPCs) (Hämmerle and Tejedor, 2002; Yabut et al., 2010). In mature neurons, DYRK1A is present in the nucleus, cytoplasm, and dendrites, with strong colocalization with synaptic clusters (Martí et al., 2003; Wegiel et al., 2004). In both DS (Altafaj et al., 2001; Nguyen et al., 2018) and AD (Ferrer et al., 2005; Kimura et al., 2007; Wegiel et al., 2011b), DYRK1A is overexpressed and accumulates across these cellular compartments. The zinc-finger transcriptional repressor REST (Lu et al., 2011), the myocyte-specific enhancer factor 2D (MEF2D) (Wang et al., 2017) or DCAF7/WDR68 (Yousefelahiyeh et al., 2018) regulate DYRK1A’s transcription; however, they operate primarily during neuronal differentiation, rather than after. DYRK1A’s widespread distribution across the neuron highlights how multiple neuronal processes can be affected by its dosage imbalance. Indeed, genes operating in vesicle exocytosis, neurotransmitter (NT) homeostasis, dendritic arborization and neuronal projections are all dysregulated by this imbalance (Brault et al., 2021). In the following sections, we summarize the functions and pathology of DYRK1A in neurons.

### 2.1 DYRK1A regulates neuronal morphogenesis and connectivity

DYRK1A mediates neuritogenesis, dendritogenesis and synaptogenesis, contributing to neuronal morphogenesis and connectivity (Manubens-Gil et al., 2023). Disruptions in these processes lead to the intellectual disabilities observed in DS individuals (Stagni and Bartesaghi, 2022). Neurons derived from DS mouse models show altered dendritic morphology, impaired axon elongation, and reduced synaptogenesis *in vitro* (Martinez de Lagran et al., 2012), mainly due to defects in chromatin-remodeling mechanisms (Lepagnol-Bestel et al., 2009). Interestingly, one study reported that DYRK1A overexpression enhanced synaptogenesis, a discrepancy likely arising from differences in the cortical regions and dendritic compartments analyzed in this study (Thomazeau et al., 2014). *In vivo*, however, DYRK1A overexpression disrupts the dendritic arborization in hippocampal neurons, reducing neuronal network activity and altering the excitation-inhibition balance in the brain (Manubens-Gil et al., 2023).

Reduced DYRK1A dosage also negatively impacts neuronal morphogenesis. Knockdown of DYRK1A *in vitro* produces neurons with shorter dendrites and fewer axons (Scales et al., 2009). In patients carrying loss-of-function DYRK1A mutations linked to autism spectrum disorder (ASD), dendritic length, branching, and synaptogenesis are all impaired (Dang et al., 2018). Similarly, in a DYRK1A haploinsufficient mouse model, cortical neurons display reduced dendritic complexity and fewer synaptic spines (Benavides-Piccione et al., 2005). Complete deletion of DYRK1A further decreases neuronal connectivity, reducing brain size and mass, defects associated with impaired growth factor signaling pathways (Levy et al., 2021).

Mechanistically, DYRK1A contributes to neuronal architecture through interactions with cytoskeletal elements (Figure 3B). In a *Drosophila* melanogaster model, DYRK1A phosphorylates β-tubulin to inhibit microtubule polymerization, a function conserved in mammals; flies carrying DYRK1A mutations exhibit defective dendritic morphology (Ori-McKenney et al., 2016). DYRK1A also phosphorylates the Neural Wiskott–Aldrich Syndrome Protein (N-WASP), suppressing Actin polymerization and filopodia formation in fibroblasts, while overexpression of a mutant, non-phosphorylatable form of N-WASP reduces synaptogenesis in neurons (Park J. et al., 2012). In addition, DYRK1A inhibits the Actin-binding LIM (ABLIM) proteins, thereby limiting cytoskeletal stabilization (Schneider et al., 2015). Through the “priming” of the GSK3β-phosphorylation target MAP1B, DYRK1A influences microtubule stability, growth cone navigation, and dendritic growth; its knockdown in cortical neurons alters neurite outgrowth *in vitro* (Scales et al., 2009). Finally, in postmortem AD and DS brain tissues, the association of DYRK1A with β-Tubulin and α-Actin is diminished, with the most potent effects observed in newborn and infant DS cases (Dowjat et al., 2012, 2019).

### 2.2 DYRK1A tunes synaptic transmission

DYRK1A orchestrates synaptic functioning by phosphorylating multiple synaptic proteins. In the presynaptic terminal (Figure 3C), DYRK1A modulates NT release by phosphorylating key proteins of the synaptic vesicle docking and fusion process, including VAMP2, SNAP25 (Guedj et al., 2012; Ouyang et al., 2023) and Munc18-1 (Park J. H. et al., 2012), although the phosphorylation of the latter does not appear to affect synaptic transmission (Classen et al., 2020). By phosphorylating MAP1A, MAP2, AP180, and α/β-adaptins, DYRK1A contributes to clathrin-mediated vesicle coating (Murakami et al., 2009) and uncoating (Murakami et al., 2012). It also targets Dynamin 1, Amphiphysin 1, Synaptojanin, and Endophilin 1, which are essential for vesicle recycling (Adayev et al., 2006; Chen C. K. et al., 2014; Chen-Hwang et al., 2002; Murakami et al., 2006; Murakami et al., 2006, 2009) and the maintenance of the synaptic vesicle pool (Geng et al., 2016). Consistently, DYRK1A overexpression disrupts vesicle recycling (Kim et al., 2010). In neurons differentiated from DS patient-derived iPSCs, normalizing DYRK1A levels restored the expression of several presynaptic proteins—including Synaptotagmin 1/3, Contactin-associated protein, Secretory Carrier Membrane 5, Endophilins (SH3GL2 & SH3GL3), Synaptic Vesicle Glycoprotein, and Intersectin 1—thereby improving vesicle dynamics and NT release (Wu et al., 2022). Interestingly, DYRK1A overdosage also impairs a presynaptic, non-NMDA type of synaptic plasticity, partly through chromatin remodeling dysregulation, leading to deficits in spatial learning and recognition memory (Lepagnol-Bestel et al., 2025).

In the postsynaptic terminal (Figure 3D), DYRK1A modulates neuronal electrophysiology and long-term potentiation (LTP), the long-lasting strengthening of synapses following strong or repeated activation (Bin Ibrahim et al., 2022). DYRK1A interacts with the subunits of the N-methyl-D-aspartate receptor (NMDAR), impacting its electrophysiological properties. DYRK1A overexpression blocks the internalization of GluN1 and GluN2A subunits, increasing the receptor’s gating and current density (Grau et al., 2014). DYRK1A is also found in protein complexes containing the subunit GluN2B and PSD95, a scaffolding protein crucial for the membrane anchorage and functioning of the NMDAR (Brault et al., 2021). These interactions suggest that DYRK1A influences ion homeostasis, and indeed, its dosage imbalance alters the neuron’s electrophysiological activity (Ori-McKenney et al., 2016). Additionally, DYRK1A regulates LTP and synaptic plasticity by modulating CaMKII phosphorylation (Brault et al., 2021; Lisman et al., 2012; Wang, 2008; Yasuda et al., 2022) at both pre- and postsynaptic terminals (Nguyen et al., 2018; Souchet et al., 2014; Thomazeau et al., 2014). Interestingly, DYRK1A overexpression reduces CaMKII phosphorylation, impairing synaptic transmission and LTP (Souchet et al., 2014; Thomazeau et al., 2014). Further LTP modulation occurs when DYRK1A phosphorylates GSK3β (Kelly and Rahmani, 2005) or interacts with proteins of the MAPK/ERK pathway (Impey et al., 1999; Kelly and Rahmani, 2005).

DYRK1A regulates NT availability by controlling its packaging into synaptic vesicles. In excitatory neurons, DYRK1A modulates the expression of the vesicular glutamate transporter (VGLUT) (Montana et al., 2004; Figure 3C, top). In overexpression models, genetic normalization of DYRK1A restored VGLUT levels (García-Cerro et al., 2018) and improved working and reference memory performance (García-Cerro et al., 2014). Interestingly, DYRK1A has a similar function in inhibitory neurons. In DS mice, DYRK1A overdosage reduced neuronal firing rates and gamma oscillations in the prefrontal cortex, associated with lower vesicular γ-aminobutyric acid (GABA) transporter (VGAT) expression and impaired GABA loading into inhibitory synaptic vesicles (Ruiz-Mejias et al., 2016; Saito et al., 2010; Shih et al., 2023; Figure 3D, bottom). Reduced inhibitory tone leads to hyperactivity and memory deficits (Cisternas et al., 2020; Jiménez-Balado and Eich, 2021). Interestingly, increased DYRK1A dosage also promotes GABAergic pathways by elevating glutamate decarboxylase 67 (GAD67) expression, although this is driven by a shift during neuronal differentiation toward a GABAergic lineage (Souchet et al., 2014).

### 2.3 DYRK1A-induced Aβ and tau neurotoxicity

When overexpressed, DYRK1A aberrantly phosphorylates amyloid precursor protein (APP) and tau, activating neurotoxic pathways. The amyloid cascade hypothesis posits that Aβ deposition arises from the sequential cleavage of APP by the β- and γ-secretase complexes (Uddin et al., 2020). DYRK1A promotes APP phosphorylation, driving its secretase-dependent cleavage and inducing Aβ production (Ryoo et al., 2008; Sun et al., 2015). Additionally, in neuronal cultures and brain organoids, DYRK1A inhibition reduced the expression of several proteins essential for the axonal transport of APP-containing vesicles. DYRK1A overexpression enhanced vesicle mobilization and density (Fernandez Bessone et al., 2022). This aberrant vesicle trafficking potentially increases APP availability, which, coupled with elevated APP phosphorylation, has the capacity to promote Aβ production and plaque formation (Figure 3E).

DYRK1A mediates tau hyperphosphorylation (Azorsa et al., 2010) at multiple residues, promoting its aggregation into toxic NFTs (Gendron and Petrucelli, 2009; Kumar et al., 2015; Figure 3F). Consistently, in DYRK1A overexpression mouse models, tau is hyperphosphorylated (Ryoo et al., 2007). In neuroblastoma cells, Aβ treatment induces DYRK1A expression, which in turn enables tau phosphorylation (Kimura et al., 2007). Beyond phosphorylation, DYRK1A overexpression also increases tau expression (Qian et al., 2013; Wegiel et al., 2011b; Yin et al., 2017) and shifts tau splicing toward the 3R isoform by phosphorylating the Alternative Splicing Factor (ASF). The resulting 3R/4R tau imbalance promotes neuronal death (Shi et al., 2008; Wegiel et al., 2011b). Conversely, inhibiting DYRK1A suppresses 3R-tau expression in human neural progenitor cells and neonatal rat brains, rescuing anxiety-related behavior and memory deficits later in life (Yin et al., 2017). DYRK1A-driven tau pathology contributes to neurodegeneration not only in AD (Ryoo et al., 2007; Shukla et al., 2023; Zhu et al., 2022) but also in other tauopathies, including Pick’s disease (PiD) (Ferrer et al., 2005), progressive supranuclear palsy (PSP), corticobasal degeneration (CBD) (Shi et al., 2008), and frontotemporal dementia (FTD) (Deboever et al., 2022; Yin et al., 2017).

Dysregulation of DYRK1A is also implicated in Parkinson’s disease (PD). DYRK1A phosphorylates α-synuclein (α-syn), which facilitates its aggregation (Kim et al., 2006) and drives dopaminergic neuron loss (Stefanis, 2012; Yong et al., 2023). Furthermore, genetic studies identified the rs8126696 DYRK1A polymorphism as a risk factor for α-syn–associated dementia (Jones et al., 2012), with positive correlations to the earlier onset of sporadic PD (Cen et al., 2016; Fan et al., 2016). *In vitro*, DYRK1A overexpression causes the death of differentiated dopaminergic SH-SY5Y-derived neurons (Chiu et al., 2019). Similarly, in SH-SY5Y cells, DYRK1A phosphorylates parkin—an E3 ligase critical for the clearance of misfolded proteins and damaged mitochondria in dopaminergic neurons (Seirafi et al., 2015)—thereby suppressing its neuroprotective activity under toxic stress (Im and Chung, 2015). Paradoxically, DYRK1A haploinsufficient mice also display a reduced number of dopaminergic neurons in the substantia nigra (Martinez de Lagran et al., 2007), suggesting that both over- and under-expression of DYRK1A compromise dopaminergic neuron survival. Indeed, DYRK1A protects dopaminergic neurons during development by inhibiting caspase-9 and limiting apoptosis (Barallobre et al., 2014), although this effect occurs according to the brain’s developmental stage and the cellular context. Furthermore, DYRK1A also phosphorylates septin 4, a cytoskeletal scaffolding protein aberrantly aggregated in tau and α-syn inclusions in AD and PD, respectively. Inhibiting DYRK1A reduces septin 4 phosphorylation and aggregation, alleviating its neurotoxic effects (Mostowy and Cossart, 2012; Sitz et al., 2008).

In Huntington’s disease (HD), DYRK1A also participates in neurotoxic pathways. A non-individualized DYRK1 (DYRK1A and DYRK1B share 85% of amino acid conservation (Aranda et al., 2011)) phosphorylates the huntingtin-interacting protein Hip-1, inducing apoptosis in H19-7 cells (Kang et al., 2005), and in transgenic mice, DYRK1A overexpression disrupts the interaction between Hap-1 and DCAF7, thereby impairing hypothalamic development (Xiang et al., 2017).

## 3 DYRK1A in the physiology and pathology of the astrocyte

The expression of DYRK1A in astrocytes is also consistent. During gliogenesis, DYRK1A is expressed in glial progenitor cells (GPCs) (Osorio et al., 2023), promoting their differentiation into astrocytes (Kurabayashi et al., 2015). In mature astrocytes, DYRK1A localizes to both the cytoplasm and nucleus, often clustering in brain regions undergoing extensive astrocytic degeneration (Kida et al., 2011; Wegiel et al., 2008, 2011a). Astrocytic DYRK1A expression is elevated in DS (Altafaj et al., 2001) and abnormally increased in AD, where it’s associated with Aβ plaques (Ferrer et al., 2005). Notably, astrocytic DYRK1A expression pattern differs: in DS brains, DYRK1A appears as granular deposits dispersed throughout the cytoplasm, whereas in AD brains, it shows a more diffuse distribution (Wegiel et al., 2008). Given its widespread localization in astrocytes, DYRK1A is likely relevant to astrocyte physiology; therefore, its overexpression could potentially impair the neuroprotective capacity of these cells. While the pathological impact of DYRK1A imbalance in neurons has been well characterized, its effects in astrocytes remain far less understood. In the following sections, we integrate the available scattered evidence to hypothesize the roles of DYRK1A in astrocytes.

### 3.1 DYRK1A drives astrocyte reactivity

Astrocyte reactivity refers to the adaptive physiological changes astrocytes undergo in response to pathological stimuli (Kumar et al., 2023; Liddelow and Barres, 2017; Qian et al., 2023). While its initial onset serves a neuroprotective role (Escartin et al., 2021; Kumar et al., 2023), prolonged reactivity promotes neuroinflammation, disrupts brain homeostasis, and contributes to neuronal death and cognitive decline (Bohmbach and Henneberger, 2024; Lawrence et al., 2023). In AD, astrocyte reactivity is a consistent feature (Chun et al., 2020; Kumar et al., 2023; Price et al., 2021), often triggered by increased Aβ levels (Craft et al., 2004; Osborn et al., 2016; Pike et al., 1994), though it can also occur independently of this (Carter et al., 2012, 2019; Marutle et al., 2013; Rodriguez-Vieitez et al., 2015, 2016; Schöll et al., 2015). A key driver of this process is the phosphorylation and activation of STAT (Ben Haim et al., 2015; Ceyzériat et al., 2016; Doherty et al., 2014; Herrmann et al., 2008; Wu et al., 2020), a known substrate of DYRK1A (Kurabayashi et al., 2015; Wiechmann et al., 2003). Under physiological conditions, DYRK1A–STAT interactions regulate astrocyte differentiation during gliogenesis (Kurabayashi et al., 2015); however, when DYRK1A is overexpressed, this interaction has the potential to occur abnormally and promote excessive STAT activation in astrocytes. Accordingly, DYRK1A inhibition suppresses astrocyte reactivity and rescues cognitive deficits in AD mouse models (Ceyzériat et al., 2018; Gong et al., 2019; Lee et al., 2020; Melchior et al., 2019; Souchet et al., 2019). STAT inhibition yields similar results (Reichenbach et al., 2019), suggesting that both proteins act along a common pathway.

Intracellular Ca^2+^ dynamics are linked to DYRK1A activity. In AD brains, astrocytes exhibit increased Ca^2+^ dynamics (Delekate et al., 2014; Haughey and Mattson, 2003; Kuchibhotla et al., 2009; Sompol et al., 2017; Takano et al., 2007), which positively correlates with the upregulated expression of reactivity markers (Alberdi et al., 2013). Reducing Ca^2+^ influx attenuates this reactive response (Paumier et al., 2022; Reichenbach et al., 2018). Importantly, Ca^2+^ activates calpains (Vanderklish and Bahr, 2000), which are overexpressed in AD (Feng et al., 2011; Shields et al., 1998; Shields et al., 2000). DYRK1A is overactivated by calpain-driven proteolysis (Jin et al., 2015). Elevated calpain expression, combined with increased Ca^2+^ levels, likely amplifies calpain activity, potentially promoting DYRK1A overactivation to increase STAT phosphorylation. This mechanism was partially validated in an AD mouse model, where a proteolytically overactivated form of DYRK1A, which becomes truncated in a Ca^2+^-dependent manner and exhibits high affinity for STAT, accumulates in astrocytes. Blocking this truncation prevents astrocytic proinflammatory signaling and restores neuronal and cognitive function (Souchet et al., 2019). Consistently, both plasma and cerebrospinal fluid (CSF) from patients with AD and DS exhibit decreased full-length and increased truncated DYRK1A levels (Moreau et al., 2022). These observations suggest that DYRK1A undergoes proteolytic overactivation when overexpressed.

Furthermore, DYRK1A potentially plays a role in Ca^2+^ regulation. In zebrafish, DYRK1A regulates vascular integrity through Ca^2+^-dependent mechanisms, possibly by directly modulating Ca^2+^ flux or Ca^2+^-related proteins (Cho et al., 2019). In endothelial cells, DYRK1A deletion reduces VEGF-induced Ca^2+^ influx, acting upstream of its release from the endoplasmic reticulum (ER) (Rozen et al., 2018). Likewise, astrocytes differentiated from iPSCs derived from ASD patients with reduced DYRK1A dosage exhibit impaired Ca^2+^ mobilization, leading to diminished neuronal network activity (Allen et al., 2022). If DYRK1A regulates Ca^2+^, its overexpression could contribute to the Ca^2+^ surge observed in AD astrocytes (Delekate et al., 2014; Haughey and Mattson, 2003; Kuchibhotla et al., 2009; Sompol et al., 2017; Takano et al., 2007), establishing a positive feedback loop to prolong astrocyte reactivity over time, where elevated Ca^2+^ and sustained calpain-dependent DYRK1A overactivation continuously upregulate STAT phosphorylation while also promoting Ca^2+^ mobilization, reinforcing this loop (Figure 3G). Altogether, these observations suggest a Ca^2+^/DYRK1A-driven mechanism underlying astrocyte reactivity; however, additional experimental studies are needed to fully elucidate this mechanism and validate this hypothesis.

Finally, further investigations are necessary to identify transcription factors that differentially regulate DYRK1A expression in astrocytes and contribute to the reactive process. One candidate is E2F1, which increases DYRK1A mRNA during cell proliferation (Maenz et al., 2008)—a process that occurs during astrocyte reactivity, although in a limited manner (Sofroniew, 2020).

### 3.2 DYRK1A’s role in excitotoxicity management

Excitotoxicity is a neurotoxic process in which glutamate released from the presynaptic terminal accumulates in the synaptic cleft due to impaired astrocytic uptake, overstimulating neuronal NMDARs and inducing neuronal death (Hynd et al., 2004; Saliñska et al., 2005). In AD, reactive astrocytes exhibit a reduced capacity to uptake synaptic glutamate (Escartin et al., 2021; Ezerskiy et al., 2022; Patani et al., 2023). Although this failure has been linked with Aβ (Li et al., 1997; Pekny and Pekna, 2014) and/or tau accumulation (Kilian et al., 2017), it can also occur independently of this (Araujo et al., 2018; Chen C. et al., 2014; Garcia et al., 2010; Heneka et al., 2005; Kawatani et al., 2021; Mizuno et al., 2018; Ponroy Bally et al., 2020; Torres et al., 2018).

DYRK1A modulates the functioning of key glutamate uptake-related proteins. Synaptic glutamate clearance is primarily mediated by the Excitatory Amino Acid Transporters (EAATs) located at the astrocytic plasma membrane (Malik and Willnow, 2019). Their distribution and activity depend on intracellular Ca^2+^ levels (Canul-Tec et al., 2022; Stenovec et al., 2008) and on activation by CaMKII (Ashpole et al., 2013; Chawla et al., 2017; Underhill et al., 2015). Since CaMKII is phosphorylated and activated by DYRK1A in neurons (Atas-Ozcan et al., 2021; Souchet et al., 2014), a similar mechanism hypothetically can occur in astrocytes, promoting EAATs activation (Figure 3H, bottom). However, DYRK1A overexpression in neurons reduces CaMKII phosphorylation (Souchet et al., 2014; Thomazeau et al., 2014). If this mechanism extends to astrocytes, this reduction can decrease the activation of EAATs and impair glutamate clearance from the synapse. Supporting these observations, DYRK1A inhibition upregulates EAAT2, enhancing astrocytic glutamate uptake both *in vitro* and *in vivo* (Li et al., 2011). Although these observations hypothetically explain the astrocytic failure to uptake glutamate observed in AD, further experimental studies are required to validate these pathways.

Importantly, DYRK1A potentially promotes astrocytic glutamate exocytosis. An additional contributor to excitotoxicity is the excessive release of glutamate from astrocytes (Ding et al., 2007; Mahmoud et al., 2019; Pham et al., 2021; Rakers and Petzold, 2017). Under physiological conditions, astrocytes release glutamate as a gliotransmitter [a signaling molecule that modulates neuronal activity (Halassa et al., 2007)] via the Ca^2+^-dependent exocytosis (Malarkey and Parpura, 2008; Mielnicka and Michaluk, 2021; Parpura and Haydon, 2000; Vardjan and Zorec, 2015) of synaptic-like microvesicles (SLMVs) (Calì, 2024; Marchaland et al., 2008). Pathological stimuli, including Aβ exposure, enhance astrocytic glutamate exocytosis (Konradsson-Geuken et al., 2009; Pham et al., 2021; Pirttimaki et al., 2013; Sharma and Vijayaraghavan, 2001; Talantova et al., 2013); however, the precise mechanism regulating this process remains unclear. DYRK1A is a strong candidate to contribute to this regulation as several of its known presynaptic vesicle-phosphorylation targets—including VGLUTs, VAMP2, and SNAP23/25—are also expressed in astrocytes (Bezzi et al., 2004; Hepp et al., 1999; Maienschein et al., 1999; Montana et al., 2004). This suggests the existence of an overlapping glutamate exocytosis mechanism between neurons and astrocytes (de Ceglia et al., 2023; Goenaga et al., 2023; Figure 3H, top). Consequently, DYRK1A overexpression in astrocytes could potentially enhance astrocytic SLMVs dynamics, while decreasing the function of EAATs, leading to synaptic glutamate accumulation and excitotoxicity. Although these observations suggest an interesting hypothesis of potential therapeutic significance, it requires further experimental validation.

### 3.3 DYRK1A in the astrocytic balance of Aβ

Astrocytes manage neuronal homeostasis by uptaking and degrading Aβ (Koistinaho et al., 2004; Wyss-Coray et al., 2003), limiting plaque formation (Davis et al., 2021; Wojtas et al., 2020). However, several components of the Aβ synthesis cascade are upregulated in reactive astrocytes (Frost and Li, 2017). DYRK1A promotes APP processing by regulating its alternative splicing, which increases the production of amyloidogenic isoforms of APP (Chu et al., 2024) and enhances APP phosphorylation, which drives its secretase-dependent cleavage to produce Aβ (Ryoo et al., 2008; Sun et al., 2015).

DYRK1A further modulates Aβ synthesis by activating presenilin, the catalytic subunit of the γ-secretase complex (Ryu et al., 2010). Interestingly, astrocytic DYRK1A inhibition decreases presenilin levels while simultaneously increasing the expression and activity of neprilysin, an endopeptidase that degrades Aβ (Lee and Hoe, 2023; Yamamoto et al., 2017). Consistently, DYRK1A overexpression suppresses, whereas its inhibition upregulates neprilysin in fibroblasts (Kawakubo et al., 2017). In AD, astrocytic DYRK1A overdosage (Altafaj et al., 2001; Ferrer et al., 2005) could potentially dysregulate the Aβ production/degradation balance in astrocytes by promoting Aβ synthesis as well as reducing its degradation (Figure 3I). This imbalance could enable Aβ production and plaque deposition while increasing the intracellular accumulation of Aβ, which induces astrocyte pathology (Söllvander et al., 2018) and degeneration (Ferrer et al., 2005; Kida et al., 2011; Wegiel et al., 2008, 2011a).

Although these findings suggest that DYRK1A dosage could regulate Aβ balance in astrocytes, further experimental studies are needed to validate this hypothesis.

### 3.4 DYRK1A and astrocytic tau pathology

Tau hyperphosphorylation in neurons promotes its toxic aggregation in NFTs (Gendron and Petrucelli, 2009; Kumar et al., 2015). In contrast, the pathological effects of astrocytic tau aggregation and toxicity remain largely overlooked. Although expressed at relatively low levels (Fleeman and Proctor, 2021; Jackson et al., 2024; Kovacs, 2020; Whitney et al., 2023), tau has been consistently detected in astrocytes (Cisternas et al., 2022; Ezerskiy et al., 2022; Fiock et al., 2023; Forrest et al., 2023; Jackson et al., 2024; Kovacs et al., 2018a; Richetin et al., 2020; Shin et al., 1991; Whitney et al., 2023), where it contributes to the formation of cytoplasmic droplets that degrade toxic peroxidized lipids (Goodman et al., 2024). Importantly, astrocytic tau aggregation has been observed in several neurological conditions, such as AD (Dabir et al., 2004; Ferrer et al., 2014; Iwatsubo et al., 1994; Nakano et al., 1992; Nolan et al., 2019), in tauopathies like CBD, PiD, PSP, FTD and argyrophilic grain disease (AGD) (Botez et al., 1999; Briel et al., 2021; Dabir et al., 2004; Feany and Dickson, 1995; Ferrer et al., 2014, 2015; Forman et al., 2005; Iwatsubo et al., 1994; Jackson et al., 2024; Komori, 1999; Kovacs, 2017; Nakano et al., 1992; Nolan et al., 2019; Sakai et al., 2006), and in aged primates (Rodríguez-Callejas et al., 2023). In these contexts, astrocytes undergo pronounced morphological alterations (Dabir et al., 2004; Komori, 1999; Kovacs et al., 2016; Nolan et al., 2019; Rodríguez-Callejas et al., 2023) reflecting a disease-specific cytoskeletal reorganization (Feany and Dickson, 1995; Ferrer et al., 2014). Importantly, tau aggregation is detrimental to astrocytic function, as it diminishes their ability to buffer reactive oxygen species (ROS)-mediated damage (Goodman et al., 2024; Hallmann et al., 2017), disrupts the expression of key homeostatic proteins, including EAAT2, aquaporin-4, and connexin 43 (Kovacs et al., 2018b; Ortiz et al., 2024), and undermines the astrocyte’s synaptoprotective capacity (Briel et al., 2021; Tanaka et al., 2024).

It has been proposed that tau aggregation in astrocytes originate from phosphorylated tau uptaken from diseased neighboring neurons (Giusti et al., 2024; Kovacs, 2020; Reid et al., 2020). However, astrocytes produce phosphorylated tau when exposed to Aβ *in vitro* (Chiarini et al., 2017), and tau aggregation in astrocytes occurs independently of neuronal tau pathology (Bachstetter et al., 2021; Ferrer et al., 2018; Kovacs et al., 2018a; Tanaka et al., 2024). Notably, several *in vivo* studies have validated the independent aggregation of astrocytic tau. In a *Drosophila melanogaster* model, astrocyte-specific tau overexpression results in its phosphorylation and accumulation in astrocytic fibrillary tangles (AFTs), which are homologous to NFTs (Colodner and Feany, 2010). Similar findings have been reported in mice, where astrocytic tau overexpression leads to its aggregation, resulting in reduced glutamate uptake. However, endogenous tau was not detected in these analyses (Dabir et al., 2006; Forman et al., 2005), likely due to its low or restricted expression. In an AD mouse model, astrocytic tau overexpression induces its aggregation, reducing the number of inhibitory synapses and parvalbumin-positive neurons, and impairing spatial memory (Richetin et al., 2020). Likewise, overexpression of 4R-tau exclusively in astrocytes promoted its aggregation and the transcription of astrocyte reactivity-associated genes, driving astrocytes toward a neurotoxic phenotype (Ezerskiy et al., 2022). Conversely, ablating tau in astrocytes prevented their Aβ-induced toxic turnover, shifting them toward a neuroprotective phenotype characterized by the secretion of synaptoprotective factors and the preservation of synapses (Cisternas et al., 2022).

Together, these findings suggest that astrocytic tau acquires pathological properties independently of neuronal tau pathology, thereby affecting astrocyte physiology. Nonetheless, the mechanism driving astrocytic tau pathology remains to be elucidated. Given the ability of overexpressed DYRK1A to pathologically phosphorylate tau in neurons, its overdosage in astrocytes could represent an overlooked contributor to astrocytic dysfunction through a similar aberrant tau hyperphosphorylation and aggregation process (Figure 3J). The experimental exploration of this hypothesis opens the opportunity to reveal novel mechanisms that could explain the loss of astrocyte-mediated neuroprotection in the AD brain.

## 4 Conclusion

DYRK1A is a dosage-sensitive pleiotropic kinase operating in the neuron–astrocyte axis, controlling neuronal morphogenesis and synaptic transmission in neurons, and potentially modulating astrocyte reactivity and excitotoxicity in astrocytes. On the other side, DYRK1A overexpression is detrimental and drives tau and Aβ pathology in neurons and possibly in astrocytes, accelerating neuronal loss and AD progression in both DS and euploid adults. However, the timing and localization of this dosage imbalance can differ between these two conditions. In DS, trisomy 21 produces lifelong DYRK1A overexpression, evident from early development and sustained across neuronal and astrocytic populations, which disrupts neurodevelopment and predisposes to premature neurodegeneration (Duchon and Herault, 2016; Soppa et al., 2014; Yin et al., 2017). In sporadic AD, by contrast, DYRK1A upregulation arises later, is localized to neurons neighboring Aβ plaques and reactive astrocytes, and could represent a secondary response rather than a constitutive imbalance (Souchet et al., 2019). Thus, DYRK1A overexpression in DS involves an early, systemic burden, whereas in AD reflects a later, region-specific, pathology-driven increase. These differences appear to account for the distinct onset, progression, and therapeutic responses observed in patients with this disease.

Nevertheless, the strong association between DYRK1A overdosage and neurodegeneration has driven efforts to develop pharmacological inhibitors of this kinase as a therapeutic strategy to mitigate AD progression. Encouragingly, in AD and DS animal models, DYRK1A inhibition in neurons reduces Aβ load and prevents tau hyperphosphorylation, while in astrocytes, it decreases astrocyte reactivity, which in turn reduces cognitive damage (Araldi and Hwang, 2023; Ballesteros-Álvarez et al., 2023; Gong et al., 2019; Lee et al., 2009, 2020; Liu et al., 2023; Melchior et al., 2019; Saleh et al., 2021; Souchet et al., 2019, 2022). Notably, neuroprotective effects have also been reported using DYRK1A inhibitors to treat neurological impairments in DS individuals (De la Torre et al., 2014; de la Torre et al., 2016). These findings support the notion that the selective, cell-specific inhibition of DYRK1A could be a promising therapy for AD; however, substantial pharmacological barriers persist in the process for identifying DYRK1A inhibitors, including biochemical properties like limited solubility, subpar blood-brain barrier (BBB) permeability, poor specificity and selectivity, and compliance with long-term safety (Bei et al., 2022; Jarhad et al., 2018; Meine et al., 2018; Powell et al., 2022). Addressing these obstacles is crucial for advancing DYRK1A inhibitors as neuroprotective agents.

Importantly, when using a pan-inhibitor, the systemic suppression of DYRK1A entails significant risks. Reduced DYRK1A dosage/activity causes neurodevelopmental syndromes (Duchon and Herault, 2016), which emphasizes potential central nervous system (CNS) liabilities associated with this approach. Additionally, systemic DYRK1A inhibition inevitably produces off-target interruptions of DYRK1A-dependent pathways outside the CNS, such as NFAT-dependent immune and cardiac signaling (Grebe et al., 2011), vascular homeostasis and angiogenesis (Takano et al., 2007), alteration of β-cell proliferation and metabolism (Shen et al., 2015), and disruption of the cell cycle, increasing cancer risk (Soppa et al., 2014). These hazards underscore the need for the identification of highly selective and CNS-targeted inhibitory compounds.

Finally, although the pathophysiology of DYRK1A has been extensively characterized in neurons, it remains largely understudied in astrocytes. This gap is critical, as astrocytes are indispensable for neuronal homeostasis and viability. When pathologically impaired, astrocytes lose their neuroprotective functions, leaving neurons vulnerable, which compromises their survival. Thus, examining the pathological consequences of DYRK1A overexpression in astrocytes could reveal novel mechanisms underlying the neurodegenerative cascade in AD. To advance this area of research, genetic models such as *Drosophila melanogaster* provide a tractable, cost-effective, quantifiable, and rapid *in vivo* system for evaluating disease phenotypes and pharmacological treatments, thereby reducing the need for costly early-stage model systems. Using the GAL4 expression system (Duffy, 2002), researchers can direct the overexpression of DYRK1A in any cell type, evaluating outcomes in a whole-organism context, in developmental and/or adult stages (Holsopple et al., 2023; Rand et al., 2023). Alternatively, *in vitro* systems resembling the neuron-astrocyte axis, like the sandwich-type neuron-astrocyte co-cultures (Kaech and Banker, 2006) offer a powerful tool to analyze the influence of astrocytic DYRK1A-overexpression on neuronal morphogenesis, and synaptic organization and function (Cisternas et al., 2016). Similarly, cerebral organoids (CO) generated from iPSCs with an abnormal number of DYRK1A gene copies provide a way of modeling neurodevelopmental defects. Although limitations exist in the current CO technology, optimized COs can facilitate the evaluation of the effects of DYRK1A-overexpressing astrocytes on neurons in a whole-brain-type context (Çağlayan, 2016). Overall, the insights gained from investigations using these and additional approaches can be leveraged to advance the rational design of next-generation DYRK1A inhibitors with enhanced potency, precision, and efficacy, specifically tailored to target DYRK1A pathological interactions within both neurons and astrocytes.
